# Social Policies and Families in Stress: Gender and Educational Differences in Work–Family Conflict from a European Perspective

**DOI:** 10.1007/s11205-016-1344-z

**Published:** 2016-05-05

**Authors:** Natascha Notten, Daniela Grunow, Ellen Verbakel

**Affiliations:** 1 Department of Sociology, Radboud University, P.O. Box 9104, 6500 HE Nijmegen, The Netherlands; 2 Department of Social Sciences, Goethe University, Theodor-W.-Adorno-Platz 6, 60629 Frankfurt am Main, Germany

**Keywords:** Work–family conflict, Gender and educational differences, Social policy, Cross-national

## Abstract

In modern welfare states, family policies may resolve the tension between employment and care-focused demands. However these policies sometimes have adverse consequences for distinct social groups. This study examined gender and educational differences in working parents’ perceived work–family conflict and used a comparative approach to test whether family policies, in particular support for child care and leave from paid work, are capable of reducing work–family conflict as well as the gender and educational gaps in work–family conflict. We use data from the European Social Survey 2010 for 20 countries and 5296 respondents (parents), extended with information on national policies for maternity and parental leave and child care support from the OECD Family Database. Employing multilevel analysis, we find that mothers and the higher educated report most work–family conflict. Policies supporting child care reduce the level of experienced work–family conflict; family leave policy appears to have no alleviating impact on working parents’ work–family conflict. Our findings indicate that family policies appear to be unable to reduce the gender gap in conflict perception and even widen the educational gap in work–family conflict.

## Introduction

Most modern societies are characterized by a considerable proportion of dual-earner couples combining work and family obligations resulting in work–family conflict (Gornick and Meyers 2003; McGinnity and Whelan 2009). Work–family conflict has important detrimental effects; including unauthorized absence from work, low levels of job satisfaction, decreases in psychological and physiological well-being as well as decreased child development (Allen et al. 2000; Barling et al. 2005; Gottfried et al. 2002). Tensions have been found to be greater for parents compared to non-parents as family demands in terms of time and effort severely rise after the transition to parenthood (Craig and Bittman 2008; Gallie and Russell 2009; Bianchi and Milkie 2010; Gauthier and Furstenberg 2002). In stimulating both women’s labour market participation and fertility, modern welfare states seek to resolve the tension between employment and care demands by policies facilitating work–family reconciliation (Grönlund and Öun 2010; Misra et al. 2011; Saraceno and Keck 2011). Family policies supporting a dual-earner/dual-carer model, such as child care services and parental leave programmes, may reduce the pressure that results from combining work and care since they facilitate outsourcing of caring tasks or (temporarily) reduce the efforts usually put into paid work. Existing national family policy packages vary markedly in their level and type of support to families. What is unclear is how effective they are, and whether existing policies indeed reduce the ‘care pressure’ for different social groups alike (Meagher and Szebehely 2012; Van Lancker and Ghysels 2012). This study sets out to examine the relationship between family policies and parents’ perceived work–family conflict along gender and educational differences in 20 European countries.

Since a few years, comparative studies on work–family conflict appear in the literature and many put forward the assumption that generous family policies will alleviate work–family conflict (e.g., Crompton and Lyonette 2006; Gallie and Russell 2009; Grönlund and Öun 2010), using child care arrangements and parental leave programmes as relevant examples. However, direct tests of the policy-hypothesis are limited (see Steiber 2009; Stier et al. 2012 for an exception) and findings remain ambiguous. The majority of studies simply assessed differences in levels of work–family conflict between a limited number of countries and some used regime types to characterize and group countries. Our study’s objective is to test the family policy hypothesis by evaluating the effects of concrete policies on work–family conflict, contributing to prior research by explicitly focusing on different social groups in a large sample of countries. This approach has appeared fruitful in studies on employment outcomes (e.g., Misra et al. 2011) and the division of labour (e.g., Bühlmann et al. 2010). We draw from important insights provided by previous studies that suggested that policies are embedded in a cultural, historical and structural context (Saraceno and Keck 2011). This implies that generous family policies will be more likely in certain contexts; for instance, extensive child care is more likely in countries with a tradition of more egalitarian gender norms (Esser and Ferrarini 2010; Steiber 2009; Strandh and Nordenmark 2006). Hence, in such contexts relationships between family policies and work–family conflict may be spurious. We argue that controlling for regime types is a sensible approach to reduce the risk of a spurious relationship. Regime types can be considered crude categorizations of countries’ policy contexts that also reflect prevailing norms, gender relations, labour market structures and historical policy traditions. As such, controlling for regime types will filter out a broad package of possibly confounding factors.

Care demands arising in families impact differently on individual resources and life chances across gender and social classes (Saraceno 2010). In particular, we are interested in differences by gender and education. Work and family spheres were traditionally highly gender segregated. The increased female labour force participation has reduced (though not eliminated) male dominance in the work domain, but responsibility for the family domain still largely rests on women (Van der Lippe and Van Dijk 2001). Educational groups differ in progressive attitudes about the division of work and care, in career and family aspirations, and in the types of jobs they possess (e.g., Davis and Greenstein 2009). These normative and structural differences may suggest that higher educated individuals perceive more pressure and stress, but at the same time one could argue that higher educated individuals have more financial, social, and cognitive resources that could help reducing work–family stress (Bianchi and Milkie 2010; Williams 2010). They may be the ‘income-rich’ but ‘time-poor’ (McGinnity and Calvert 2009). We would like to complement this argument by suggesting that family policies potentially mitigate or intensify gender and education effects. First, family policies have a much stronger effect on mothers’ employment as compared to fathers’ employment. Second, previous studies of work–family conflict largely fail to acknowledge that specific family policies address work family conflict in distinct ways, namely by either reducing parents’ time at work or by facilitating both parents’ continuous employment. Reduced working hours and extended time at home, often accompanied by a reduced income, may alleviate work–family conflict for new parents, but only for those who can economically afford earning a reduced income. Extensive child care policies, in contrast, facilitate continuous paid work for all parents and are therefore probably better suited to address work–family conflict among those with lower educational and labour market resources. We therefore think that gender and education are interesting social cleavages to study in terms of work–family conflict, and to examine whether policies are capable of diminishing these cleavages. Assessing whether policies are equally effective for different groups of parents may increase our understanding of the workings of child care and parental leave programmes. Because we are interested in the effects of policies aimed at working parents, we restrict our sample to dual-earner parents, including those on family leave.

This study sets out to answer the following three research questions: (1) *To what extent does the level of work*–*family conflict among parents differ by gender and educational level?* (2) *To what extent do child care services and parental leave programmes influence parents’ perceived work*–*family conflict?* (3) *Do child care services and parental leave programmes moderate gender and educational differences in parents’ perceived work*–*family conflict?*

Work–family conflict might result in more stress at home, but may also have an impact on the work place (Higgins et al. 1992; McGinnity and Russell 2013). We therefore study two related but distinct dimensions underlying the overall concept of work–family conflict (Amstad et al. 2011; Byron 2005; Mesmer-Magnus and Viswesvaran 2005): conflict due to work demands interfering with family life (work-to-family conflict) and conflict related to the interference of family demands with work performance (family-to-work conflict). In doing so, we aim to contribute to a small but growing literature investigating effects of national policies on both types of strain (Ruppanner and Huffman 2013). Specific family policies may address the two distinct aspects of work–family conflict differently. For instance, by providing long and generously paid parental leaves, welfare states grant parents the opportunity to stay at home with their children for extended periods. Hence, generous parental leave should temporarily reduce work-to-family conflict but not family-to-work conflict. In contrast, by offering affordable high-quality day care for children under the age of three, welfare states enable new parents to remain continuously employed upon entering parenthood. This strategy should help parents deal with family-to-work conflict but not with work-to-family conflict.

We will draw from role stress theory to derive hypotheses about gender and educational differences in work–family conflict. To empirically test our individual-level, country-level, and cross-level interaction hypotheses, we make use of data from the European Social Survey (ESS) 2010. We conduct multilevel analyses on 5269 respondents (i.e., working parents) from 20 countries. Country-level information on the family policies of interest—child care and parental leave—comes from the OECD Family Database (2012). Note that true causal inferences cannot be made on the basis of this cross-sectional design.

## Theoretical Background and Hypotheses

Work–family conflict is argued to originate from interrole conflict. Participating in both work and family domains implies participation in different roles; if pressures that result from one role compete with pressures that result from another role, people are likely to experience work–family conflict. Reasoned from the basic assumption that time and energy to fulfil roles are scarce, role conflict may emerge from time-based conflict, strain-based conflict, and behaviour-based conflict (Greenhaus and Beutell 1985; Peters et al. 2009). Time-based conflict implies that time spent on one role cannot be spent on another role; for instance, the time needed for commuting and working cannot be used for child care. Strain-based conflict refers to strain (such as tension or fatigue) produced by one role hindering one’s performance in another role; for instance, having an ill child or marital problems may reduce one’s ability to concentrate on work. Behaviour-based conflict arises if behavioural patterns that are common or needed in one role are incompatible with behaviour expected in another role; for instance, switching from being the strong and coercing manager at work to the emotional and caring person at home produces strain.

Due to these different forms of conflict, combining work and family responsibilities increases parents’ stress levels in daily life, resulting in lower levels of well-being, health, and satisfaction related to both the work domain (e.g., burnout) and family domain (e.g., marital satisfaction) (Allen et al. 2000; Amstad et al. 2011). Note that the combination of work and family roles is also argued to have positive spill-over effects from one role to the other because multiple roles create more opportunities and higher levels of satisfaction (Greenhaus and Powell 2006; Grönlund and Öun 2010). The aim of this study is to analyse policy implications for different groups of parents in their experienced work–family conflict, which may contribute to gaining insights in social inequalities in subsequent potential negative and positive outcomes of work–family conflict.

### Gender Differences

Both time-based and strain-based conflicts lead us to expect that working mothers experience higher levels of change in work–family conflict than working fathers (Hypothesis 1). Firstly, women are more engaged in family care and household work than are men (Bianchi et al. 2000; Stier and Lewin-Epstein 2007). As a result, they experience more dramatic changes in both, their work and family time after the transition to parenthood (Kühhirt 2012; Schober 2013). Especially in countries with limited access to part-time work, women spend more total time working as compared to men (United Nations 2010). In countries with more employment flexibility new mothers often reduce work hours or take up less demanding jobs as a reaction to work–family conflict (Gash 2009). These adaptation processes result in gender-balanced patterns of total time working in the U.S. and the rich northern European countries when paid and unpaid work are summed up (Burda et al. 2007).

Secondly, gendered norms about family care increase the experience of role strain of women more than that of men (Poortman and Van der Lippe 2009; Stier and Lewin-Epstein 2007). The strength of gender norms varies, but generally, mothers are more strongly expected than fathers are to take up the largest share of caring and household tasks. The accompanying pressure to perform in the family role next to the work role makes working mothers more vulnerable to experience time pressure and role strain. Accordingly, most studies on this issue demonstrate that women experience more work–family conflict than men once working hours are controlled for (Crompton and Lyonette 2006; Grönlund and Öun 2010; Offer and Schneider 2011; Steiber 2009; Stier et al. 2012; Strandh and Nordenmark 2006). Role pressures are also likely to vary in strength across countries. After controlling for individual-level factors associated with work–family conflict Steiber (2009) found evidence of greater conflict in more gender egalitarian and more affluent countries. Note that since our study is based on working parents, we are likely to observe a share of mothers that have already adapted their employment as a response to work–family conflict. As a consequence, observed gender differences in work–family conflict will be underestimated rather than overestimated.

### Educational Differences

The reasoning behind the different types of conflict leads us to expect that highly educated employed parents experience higher levels of work–family conflict than poorly educated employed parents (Hypothesis 2). Gallie and Russell (2009) demonstrated that jobs that require higher skill levels, more responsibility and that are characterized by high levels of work intensity increase work–family conflict. On average, highly educated employees hold more ‘demanding’ jobs than do poorly educated employees (Van der Lippe et al. 2006). These demanding jobs require working extra hours and more unsocial working hours, such as in evenings or weekends (Gallie and Russell 2009). This is time that cannot be spent at family tasks and induces time-based conflict. At the same time, demanding jobs make it harder to cut oneself of work; issues from work are more likely to be taken home, hindering the performance of the caring role. Strain-based conflict may also result from mental fatigue that emerges from cognitive challenges that are caused by the complexity of the tasks in those demanding jobs (Gallie and Russell 2009). Note that some argue that employee flexibility, which is more prevalent among jobs occupied by highly educated, may help to accommodate the combination of work and care; however this does not appear to offset the negative effects of higher work pressure (Gallie and Russell 2009; McGinnity and Calvert 2009).

Besides job characteristics of the higher educated, their normative expectations and social context are argued to intensify their work–family conflict compared to the lower educated. In contrast to traditional gender norms that promote a male breadwinner and female carer role, progressive gender norms emphasize that both men and women should combine work and care (Van der Lippe et al. 2006). The pressure that results from the expectation to perform in both work and family role will induce strain-based conflict (Steiber 2009). Since progressive gender norms are mainly embraced by highly educated persons and their contexts (e.g., Bolzendahl and Myers 2004; Thornton et al. 1983), we expect that higher educated employees suffer more strongly from work–family conflict than low educated employees. Finally, pressure to perform in the work role is also likely to be higher among higher educated relative to low educated because the former find it more important to pursue a career; high ambition levels are accompanied by a constant pressure to excel (Grönlund and Öun 2010; McGinnity and Calvert 2009).

### The Impact of Family Policies

Different social contexts, partly characterized by different national family policies, may affect the level of work–family conflict people experience (Gornick and Meyers 2003; Strandh and Nordenmark 2006; Van der Lippe et al. 2006). Aiming at increasing female labour market attachment and fertility, governments introduce family policies that are designed to facilitate the combination of work and care (Misra et al. 2011). Earlier studies (e.g., Strandh and Nordenmark 2006; Van der Lippe et al. 2006; Stier et al. 2012) suggest family policies to have alleviating effects on work–family conflict as facilitating the combination of work and family is their main objective. However, research also puts forward that countries offering extensive day care facilities for children below age 3 have on average higher reports of work–family conflict (Steiber 2009). This finding may point to individual selection into (levels and amounts of) paid work as a mediator of work–family conflict (Steiber 2009), though studies reveal contrasting viewpoints regarding this issue (Stier et al. 2012). We extend on this research in two ways.

*First*, we argue that by facilitating continued dual earning rather than homemaking, child care services reflect one particular strategy of existing family policies in Europe. The second policy strategy grants parents extended time away from their job by offering generous family leave.^1^ In general, both policy strategies reflect distinct work–family ideals that are expected to alleviate perceived work–family conflict (Hypothesis 3). However, we argue that both strategies of family supportive policies need to be considered simultaneously in an analysis of work-family stress because most welfare states offer them in various combinations and both types of family policies function to some degree as substitutes (Fig. 2). Early public childcare services are currently best developed in the social democratic welfare states. Early public childcare also used to be widespread in the eastern European countries under communism though the quality has been perceived very poor. Against this background, use and availability of public childcare almost disappeared in post-communist countries while extended family leaves are nowadays widespread. In general, family leaves appear to have an inverse u-shaped effect on female labour force participation. Absence of legal family leaves withholds parents (and especially mothers) from being active on the labour market and also very long leaves seem to discourage female labour market participation, probably because long-term leaves erode human capital and career chances in the long run (Bergmann 2009; Edin and Gustavsson 2008; Misra et al. 2011; Ray et al. 2010). The policy strategy of very long family leave is historically rooted in the conservative welfare regimes and is often accompanied by other policy measures that favour a main-breadwinner model in families. In these countries, financial compensation during the leave is usually low while the leave length is generous and foresees a job guarantee for the parent on leave. The Scandinavian countries, in comparison, offer shorter parental leaves but these leaves are much better paid. The shortest family leaves in Europe can be found in the liberal welfare states, in Great Britain but also in Ireland and Belgium.

*Second*, we argue that these various types of family policies are likely to affect different social groups in different ways. More specifically, we hypothesize that distinct national family policies mitigate or intensify existing gender and educational differences in work–family conflict. For one, both types of family policies are expected to more strongly reduce mothers’ perceptions of work–family stress as compared to fathers’ perceptions (Hypothesis 4). Family policies most strongly intervene in actions that are typically part of the mothers’ role, i.e. taking care of children and the home; they are therefore often labelled as ‘women friendly’ (see e.g., Stier and Lewin-Epstein 2007). Consequently, we expect women to be more susceptible to family policies than men, which would reduce the size of the gender gap in work–family policies.

With respect to educational differences, we expect the two policy types to have distinct implications. Grunow et al. (2011) have found low educated mothers to take longer family leaves than highly educated mothers. At first glance, this suggests that the low educated benefit more from these leaves (cp. Mandel and Shalev 2009). However, when it comes to labour market participation in general, a recent study of the OECD countries found that highly educated women respond more often to the opportunities to combine motherhood and employment provided by reconciliation policies (Nieuwenhuis et al. 2012). Extended time at home in family leave may thus alleviate work–family conflict for parents, but, assuming that parental leave reduces family income and financial security, apparently more so for those who can economically afford it (i.e., the higher educated). As low educated women and men tend to have low educated partners, both are more likely to hold low-paid and more vulnerable jobs and thus to experience higher levels of financial instability and strain. Financial strain is associated with increasing work–family conflict in Europe (Gallie 2013). Therefore, factors such as financial strain might have exacerbating effects on work–family conflict among the low educated, even in countries where family leaves are long. Hence, when it comes to work–family conflict, leave policies might alleviate stress levels especially for the higher educated, leading to smaller educational gaps in work–family conflict. Few countries—among them the Scandinavian countries—offer parents on leave full or close to full compensation of forgone earnings. In these countries, the education effect should be weak or non-existing. Since different processes seem to be at work, which to some extent would cancel each other out, we may expect only weak interaction effects between available family leaves and educational levels (Hypothesis 5).

In contrast to leave policies, extensive child care policies facilitate continuous paid work for all parents and are therefore probably better suited to address work–family conflict among those with lower educational and labour market resources (as in less financial strains and job insecurity). Against our baseline expectation that the highly educated face more work–family conflict than the low educated, extensive child care for children below age 3 might therefore exacerbate existing differences in work–family conflict between the highly educated and the low educated (Hypothesis 6).

## Data and Methodology

We use data from the European Social Survey (ESS) round 5, 2010 (2012), which includes questions on work, family and well-being in the supplementary questionnaire. ESS employs random probability samples of the population of 15 years and older in private households and conducts face-to-face interviews. Rigorous methodological standards guarantee high comparability between countries. We selected respondents who reported holding a paid job (including those on leave) and living together with their partner and children at the time of interview. Our selection is motivated by the fact that one needs to be involved in both a work and family role in order to be at risk of experiencing work–family conflict and to be interviewed about this topic.^2^ After removing cases with missing values, information about work–family conflict is available for 6572 respondents in 23 countries. We supplemented the individual-level data with data on national policy arrangements regarding child support and maternity/parental leave derived from the OECD Family Database (2012). No country-level data were available for Cyprus, Switzerland, and Israel, leaving 5296 respondents (i.e., working parents) in 20 countries in our analyses.

### Dependent Variables

We distinguish two forms of work–family conflict: (a) ‘work-to-family conflict’, indicating the impact of work responsibilities on the family life, and (b) ‘family-to-work conflict’, representing the impact of family responsibilities on work. Prior research has shown these two concepts to be correlated but distinct in their variance, outcomes, and predictors (Amstad et al. 2011; Byron 2005; Mesmer-Magnus and Viswesvaran 2005). Factor analysis on the ESS data confirmed these two dimensions of work–family conflict.

Work-to-family conflict is measured by the following items: (a) How often do you keep worrying about work problems when you are not working? (b) How often do you feel too tired after work to enjoy the things you would like to do at home? (c) How often do you find that your jobs prevents you from giving the time you want to your partner or family? Answer categories were: (0) never (1) hardly ever (2) sometimes (3) often (4) always. A scale was constructed by taking the mean score (α = 0.67). Family-to-work conflict is measured by the following items: (a) How often do family responsibilities prevent you from giving the time you should to your job? (b) How often do you find it difficultto concentrate on work because of your family responsibilities? Again, answer categories were: (0) never (1) hardly ever (2) sometimes (3) often (4) always.^3^ A scale was constructed by taking the mean score (α = 0.72).

### Individual-Level Independent Variables

The main variables of interest are gender (female coded as 1) and educational level. Educational level refers to the highest education obtained and is measured by the ISCED score on a 7-point scale: (0) less than lower secondary, (1) lower secondary, (2) lower tier upper secondary, (3) upper tier upper secondary, (4) advanced vocational, sub-degree, (5) lower tertiary education (Bachelor degree), and (6) higher tertiary education (at least Master degree).

Furthermore, we include several basic and important determinants of work–family conflict, relating to either the work or family domain. Working hours of the respondent refer to the total hours the respondent normally works per week in his/her main job, including overtime, and was top-coded at 70 h per week. We also included individual and work-related characteristics of the partner that may be relevant for the level of work–family conflict that respondents experience. The partner’s education is measured similar to respondent’s education. Since not all partners have a job (around 20 %), we included dummy variables indicating whether the partner is not working (reference category), works <32, 32–40 h, or more than 40 h a week. The presence of children in the family home, and especially in the early years of child care, has been found to increase role conflict due to work and family demands as well (Esser and Ferrarini 2010; Stier et al. 2012; Voydanoff 2002). Presence of young children in the family home was measured by two dummy variables: at least 1 child is 3 years old or younger (reference category), and all children are between 4 and 18 years old. We also included the number of children in the family home, using dummy variables. We distinguish between one child (reference category), 2 children or three or more children living at the parents’ home.

Finally, we control for respondents’ age by using a continuous variable. Note that all continuous individual-level independent variables are centred to their means (grand mean centering).

### Policy Measures

Countries’ support of child care is represented by the fulltime equivalent participation rate of child care in formal care or pre-school for children younger than age 3 in 2008. This measure takes into account the amount of hours children spent at day care or preschool, which is sensible since the intensity of child care use varies considerably across countries (OECD Family Database 2012). Values vary from 1.14 in Czech Republic to 74.35 in Denmark (see also Table 2). Countries’ support of maternity and parental leave is measured by the full-rate equivalent of paid maternity and parental leave, 2007/2008. This measure sums paid maternity leave and paid parental leave in weeks. Because both maternity and parental leave systems vary between countries—despite European directives (Pregnant Worker 1992, Parental Leave 1996)—we make use of the full-rate equivalent, which equals duration of leave in weeks times payment (as per cent of average wage earnings) received by the claimant (OECD Family Database 2012). As presented in Table 2, values vary from 6.61 in Ireland to 85.39 in Estonia. Both country-level indicators of family policy are z-standardized over countries in the multilevel models. This policy indicator is not ideal insofar as it does not differentiate between the duration of leave and the level of payment. We ran additional analyses in which we separated the maximum leave duration and the income compensation during the leave in two separate indicators. These analyses showed negative significant effects of the level of compensation on family-to-work stress but no effect for duration. However, since the two indicators correlate by 0.6 we decided to stick with the combined measure in the analyses presented in this paper.

We control for welfare state regimes in order to filter out spurious elements in the relationship between child care and leave policies and work–family conflict that are due to historical, political, and attitudinal climates (Saraceno and Keck 2011; Steiber 2009; Strandh and Nordenmark 2006; Van der Lippe et al. 2006). Such climates will have led to specific policy measures regarding child care and leave programmes and may independently affect work–family conflict. We follow prior research on the classification of welfare state regimes that particularly focuses on family and gender policies (Arts and Gelissen 2002; Korpi 2000; Bühlmann et al. 2010) by categorizing countries as liberal welfare state regimes (Ireland, the Netherlands and the United Kingdom see Bühlmann et al. 2010), conservative regimes (Belgium, France, Germany, Greece, Portugal and Spain), social-democratic regimes (Denmark, Finland, Norway and Sweden) or as post-communist welfare regimes (Polen, Bulgaria, Croatia, Czech, Estonia, Hungary, Slovakia and Slovenia). Descriptive statistics of all variables included are presented in Table 1. See Table 2 for more detailed information on country-level characteristics and means.

**Table 1:** Descriptive statistics

	Minimum	Maximum	Mean or proportion	SD
*Individual*-*level*				
Work-to-family conflict	0	4	1.86	0.78
Family-to-work conflict	0	4	0.94	0.76
Gender respondent (1 = female)	0	1	0.45	
Educational level respondent	0	6	3.47	1.75
Educational level partner	0	6	3.36	1.76
Working hours respondent	1	70	40.80	11.20
Non-working partner (Ref.)	0	1	0.20	
Partner works <32 h	0	1	0.14	
Partner works 32–40 h	0	1	0.18	
Partner works >40 h	0	1	0.49	
Children ≤3 years at home (Ref.)	0	1	0.34	
Children 4–18 years at home	0	1	0.66	
One child at home (Ref.)	0	1	0.39	
Two children at home	0	1	0.47	
Three or more children at home	0	1	0.15	
Age respondent	19	60	39.85	7.27
*Country*-*level*				
Formal child care or pre-school (<3 years)	1.14	74.35	32.12	19.67
Maternity and parental paid leave	6.61	85.39	37.28	20.53
Liberal regime (Ref.)	0	1	0.16	
Conservative regime	0	1	0.33	
Social-democratic regime	0	1	0.24	
Post-communist regime	0	1	0.27	

*Source*: ESS 2010. N1 = 5.296, N2 = 20

**Table 2:** Country level characteristics and means

Country	Work-to-family (mean)	Family-to-work (mean)	Child care	Maternity and parental leave	Regimes
Ireland	1.46	0.86	25.85	6.61	Liberal
Portugal	1.60	0.95	59.99	17.00	Conservative
Netherlands	1.66	0.88	34.47	21.35	Liberal
Hungary	1.66	0.68	8.83	76.12	Post-communist
Norway	1.66	0.93	55.12	38.82	Social-democratic
Denmark	1.75	0.83	74.35	32.25	Social-democratic
Slovenia	1.83	0.82	40.32	52.00	Post-communist
Bulgaria	1.86	0.66	16.24	56.70	Post-communist
Spain	1.86	0.77	34.51	16.00	Conservative
United Kingdom	1.86	1.10	22.19	12.79	Liberal
Sweden	1.90	1.00	51.09	37.70	Social-democratic
Belgium	1.91	0.93	46.82	14.36	Conservative
Poland	1.94	0.97	9.29	39.10	Post-communist
Finland	1.96	1.18	32.87	35.66	Social-democratic
Germany	1.97	0.94	13.55	54.65	Conservative
France	2.01	0.81	43.40	43.78	Conservative
Estonia	2.01	1.09	21.70	85.39	Post-communist
Czech Republic	2.06	1.18	1.14	63.36	Post-communist
Slovakia	2.11	1.26	2.86	46.10	Post-communist
Greece	2.18	1.06	15.93	25.39	Conservative

*Source*: ESS 2010. N1 = 5.296, N2 = 20

### Methods and Models

We employ random intercept multilevel analyses to take account of the nested structure of individuals within countries. The null-model (not presented) demonstrates that experienced work–family conflict significantly differs across countries, although most of the variance in work–family conflict is situated at the individual level. For work-to-family conflict 5 % of the variation can be attributed to differences between countries (ICC = 0.050); for family-to-work conflict 3.8 % of the variation can be attributed to differences between countries (ICC = 0.038). For both work-to-family conflict (Table 3) and family-to-work conflict (Table 4), Model 1 includes all individual and country level variables and tests our hypotheses about differences in work–family conflict by gender and education. All models include a random intercept and a random effect of either gender or education in the interaction models, all other effects are fixed. Note that though the variance components of the random slopes of gender and education are not significant, our theoretical reasons to consider cross-level interactions allow testing these interactions (Snijders and Bosker 1999). Models 2 and 3 include cross-level interaction effects between gender and policies and between education and policies respectively to assess to what extent family policies succeed in closing gaps. Models 3a and 3b present the cross-level interaction effects between education and policies for mothers and fathers separately, to explore whether the effect of policy on education might be different for men and women.

**Table 3:** Multilevel models work-to-family conflict

	Model 1		Model 2		Model 3		Model 3a (fathers)		Model 3b (mothers)	
	b	SE	b	SE	b	SE	b	SE	b	SE
*Individual*-*level*										
Gender respondent (1 = female)	0.079**	0.025	0.077**	0.025	0.080**	0.025				
Educational level respondent	0.038***	0.007	0.038***	0.007	0.037***	0.007	0.030**	0.010	0.049***	0.012
Educational level partner	−0.006	0.007	−0.006	0.007	−0.005	0.007	−0.001	0.010	−0.009	0.011
Working hours respondent	0.021***	0.001	0.021***	0.001	0.021***	0.001	0.020***	0.001	0.022***	0.002
Non-working partner (Ref.)										
Partner works <32 h	−0.051	0.037	−0.047	0.037	−0.051	0.037	−0.018	0.042	−0.118	0.089
Partner works 32–40 h	−0.115**	0.037	−0.113**	0.037	−0.114**	0.037	−0.086	0.045	−0.158*	0.068
Partner works >40 h	−0.015	0.030	−0.014	0.030	−0.014	0.030	−0.018	0.037	−0.034	0.058
Children ≤3 years at home (Ref.)										
Children 4–18 years at home	−0.008	0.026	−0.006	0.026	−0.008	0.026	−0.010	0.034	0.002	0.040
One child at home (Ref.)										
Two children at home	0.058**	0.022	0.058**	0.022	0.058**	0.022	0.100**	0.030	0.010	0.033
Three or more children at home	0.053	0.031	0.052	0.031	0.052	0.031	0.081*	0.041	0.021	0.049
Age respondent	−0.001	0.002	−0.001	0.002	−0.001	0.002	0.000	0.002	−0.003	0.003
*Country*-*level*										
Maternity and parental paid leave	0.029	0.045	0.043	0.046	0.028	0.002	0.032	0.048	0.037	0.047
Formal childcare or pre-school (<3 years)	−0.106**	0.039	−0.111**	0.041	−0.104**	0.039	−0.117**	0.042	−0.083*	0.041
Liberal regime (Ref.)										
Conservative regime	0.190*	0.089	0.191*	0.089	0.192*	0.088	0.242*	0.094	0.128	0.091
Social-democratic regime	0.169	0.116	0.169	0.115	0.161	0.114	0.224	0.122	0.075	0.118
Post-communist regime	−0.059	0.125	−0.056	0.125	−0.058	0.123	0.021	0.133	−0.168	0.131
*Cross*-*level interactions*										
Maternity and parental paid leave × gender			−0.032	0.023						
Formal childcare or pre-school (<3 years) × gender			0.012	0.023						
Maternity and parental paid leave × education					0.007	0.007	0.017	0.009	0.001	0.012
Formal childcare or pre-school (<3 years) × education					0.013*	0.007	0.018*	0.009	0.009	0.011
Constant	1.750***	0.090	1.747***	0.090	1.750***	0.089	1.665***	0.097	1.945***	0.109
*Variance components*										
Variance gender slope			0.000	0.000						
Variance education slope					0.000	0.000	0.000	0.000	0.000	0.001
Country-level variance	0.011*	0.004	0.011*	0.004	0.010*	0.004	0.011*	0.005	0.009*	0.004
Individual-level variance	0.535***	0.010	0.535***	0.010	0.535***	0.010	0.519***	0.014	0.551***	0.016
N level 1	5296		5296		5296		2896		2400	
Log-likelihood	−5877.90		−5876.28		−5875.88		−3173.08		−2702.75	

*Source*: ESS 2010. N level 2 = 20

* *p* ≤ 0.05, ** *p* ≤ 0.01, *** *p* ≤ 0.001, two-tailed tests

**Table 4:** Multilevel models family-to-work conflict

	Model 1		Model 2		Model 3		Model 3a (fathers)		Model 3b (mothers)	
	b	SE	b	SE	b	SE	b	SE	b	SE
*Individual*-*level*										
Gender respondent (1 = female)	0.068**	0.025	0.070**	0.026	0.068**	0.025				
Educational level respondent	0.037***	0.007	0.037***	0.007	0.039***	0.008	0.036***	0.010	0.046***	0.013
Educational level partner	0.008	0.007	0.008	0.007	0.009	0.007	0.007	0.010	0.008	0.011
Working hours respondent	0.008***	0.001	0.008***	0.001	0.008***	0.001	0.009***	0.001	0.008***	0.002
Non-working partner (Ref.)										
Partner works < 32 h	−0.018	0.038	−0.014	0.038	−0.019	0.037	0.021	0.042	−0.160	0.088
Partner works 32–40 h	−0.076*	0.037	−0.077*	0.037	−0.075*	0.037	−0.006	0.046	−0.256***	0.068
Partner works > 40 h	0.005	0.031	0.002	0.031	0.005	0.031	0.026	0.038	−0.129*	0.058
Children ≤3years at home (Ref.)										
Children 4–18 years at home	−0.079**	0.026	−0.077**	0.026	−0.080**	0.026	−0.054	0.035	−0.110**	0.040
One child at home (Ref.)										
Two children at home	0.081***	0.022	0.081***	0.022	0.080***	0.022	0.071*	0.030	0.093**	0.033
Three or more children at home	0.074*	0.032	0.075*	0.032	0.074*	0.032	0.081	0.042	0.078	0.049
Age respondent	−0.002	0.002	−0.001	0.002	−0.002	0.002	0.000	0.002	−0.003	0.003
*Country*-*level*										
Maternity and parental paid leave	−0.025	0.053	−0.005	0.054	−0.026	0.052	−0.011	0.057	−0.026	0.049
Formal childcare or pre-school (<3 years)	−0.102*	0.046	−0.092	0.048	−0.101*	0.046	−0.082	0.050	−0.116**	0.043
Liberal regime (ref)										
Conservative regime	0.005	0.105	0.002	0.105	0.004	0.104	−0.034	0.113	0.020	0.097
Social-democratic regime	0.157	0.136	0.158	0.136	0.146	0.135	0.135	0.147	0.125	0.126
Post-communist regime	−0.080	0.146	−0.071	0.147	−0.082	0.145	−0.040	0.159	−0.180	0.138
*Cross*-*level interactions*										
Maternity and parental paid leave × gender			−0.048	0.025						
Formal childcare or pre-school (<3 years) × gender			−0.017	0.025						
Maternity and parental paid leave × education					0.020**	0.008	0.025**	0.009	0.022	0.013
Formal childcare or pre-school (<3 years) × education					0.018*	0.007	0.019*	0.009	0.017	0.013
Constant	0.920***	0.105	0.917***	0.105	0.923***	0.104	0.880***	0.114	1.160***	0.114
*Variance components*										
Variance gender slope			0.001	0.003						
Variance education slope					0.000	0.000	0.000	0.000	0.001	0.001
Country-level variance	0.016*	0.006	0.016*	0.006	0.016*	0.006	0.017*	0.007	0.010*	0.005
Individual-level variance	0.547***	0.011	0.547***	0.011	0.546***	0.011	0.539***	0.014	0.547***	0.016
N level 1	5296		5296		5296		2896		2400	
Log-likelihood	−5940.04		−5937.88		−5934.38		−3231.19		−2697.83	

*Source*: ESS 2010 N level 2 = 20

* *p* ≤ 0.05, ** *p* ≤ 0.01, *** *p* ≤ 0.001, two-tailed tests

## Descriptive Results

**Fig. 1:**
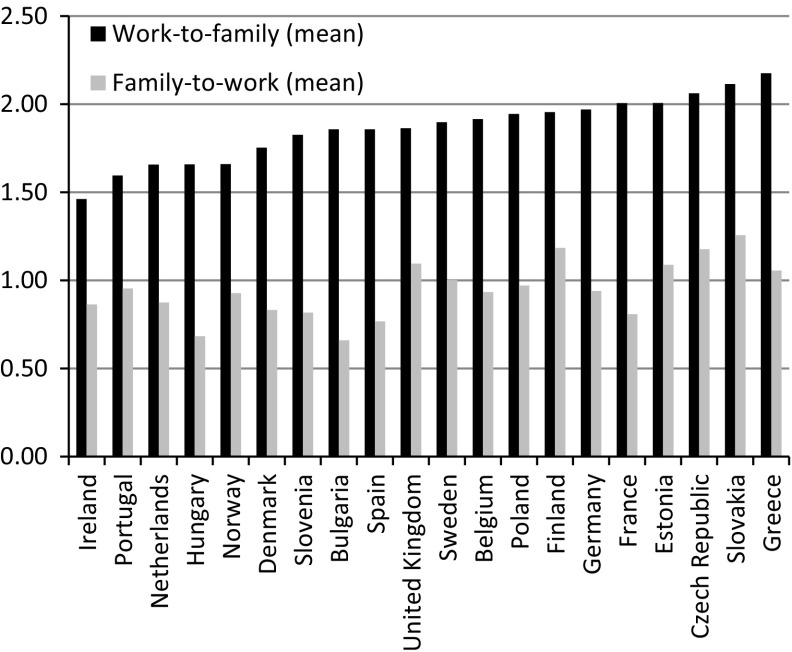
Work–family conflict; means per country

Figure 1 shows the mean level of the two dimensions of work–family conflict under study, that is, family-to-work conflict and work-to-family conflict per country (not controlling for individual and country level features, see also Table 2). In all countries, the mean level of work-to-family conflict is higher than the perceived family-to-work conflict. In Ireland and Portugal, respondents report on average the lowest level of work-to-family conflict, whereas parents in Slovakia and Greece experience on average the highest levels of work-to-family conflict. In Bulgaria and Hungary the average level of experienced family-to-work conflict is lowest, whereas the highest average levels of family-to-work conflict are found for Slovakia, Czech Republic and Finland (see also Table 2). Note that there is a significant correlation between the mean scores of both dimensions of work–family conflict across the countries under study (*r* = 0.42). Overall, we may conclude that a higher level of work-to-family conflict relates to a higher level of family-to-work conflict.

**Fig. 2:**
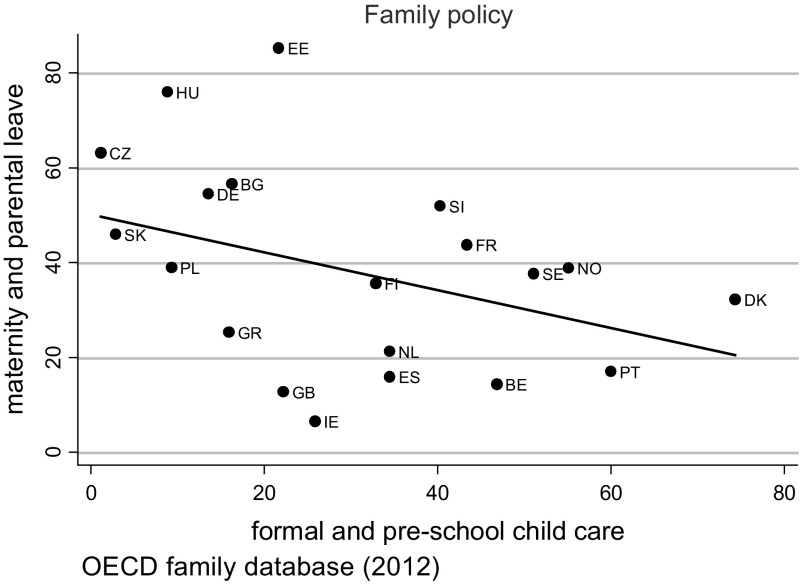
The relation between child care and leave policy

Figure 2 shows the association between the two family policies under study: support of child care and maternity/parental leave. These indicators are commonly argued to represent a country’s level of dual-earner/dual-carer policy (Gornick and Meyers 2003) and are both directed at facilitating the combination of work and care. As mentioned before, the content but also consequences of these two different family policies are complex. Figure 2 demonstrates that the two are not simply part of one and the same policy strategy and may have distinct functions: a higher level of child care relates to a lower level of leave (and vice versa). There are some outliers, for instance Ireland (IE), United Kingdom (GB) and Estonia (EE). Ireland and the United Kingdom score rather low on both, which clearly reflects liberal welfare policies. Estonia scores highest in the sample on leave provision, whereas its child care coverage is comparable to the level provided in the United Kingdom.

## Multilevel Results

### Work-to-Family Conflict

In line with our expectations (hypotheses 1 and 2), Model 1 in Table 3 shows that mothers and higher educated report higher levels of work-to-family conflict as compared to fathers and lower educated parents. The gender difference is 0.079 on a scale from 0 to 4, meaning that ‘an average father’ scores 1.75 (i.e., the constant) points on work-to-family conflict, compared to a significantly higher 1.83 for ‘an average mother’. Each subsequent step on the educational ladder increases respondents’ stress level with 0.038; the maximum impact of education amounts to 0.23 (6 × 0.038).^4^ The number of working hours is highly relevant; longer working days lead to more work-to-family conflict (*b* = 0.021). Having a working partner reduces the experienced stress due to the impact of work-issues on the family live compared to having a non-working partner, but only when the partner works between 32 and 40 h a week (*b* = −0.115). Separate analyses for both sexes (see also Models 3a and 3b) show that this effect is entirely driven by women. Thus for mothers a fulltime stay-at-home partner is more stressful than a regular working partner. There is no significant difference between the impact of young children (≤3 years old) at home versus older ones (4–18 years old) on parents’ perceived work-to-family conflict. However, parents with two children at home experience significantly more work-to-family conflict compared to parents taking care of one child at their home. Conducting the analyses for fathers and mothers separately shows that more children at home is predominantly stressful for fathers.

Model 1 also shows the effects of the two distinct family policies, controlled for countries’ regime type, as formulated in hypothesis 3. As expected (see also Stier et al. 2012), and controlled for several individual and country characteristics, policies supporting child care significantly reduce respondents’ experienced work-to-family conflict (*b* = −0.106). In contrast, policies supporting maternity and parental leave do not seem to alleviate work-to-family conflict. A robustness check revealed that including a quadratic term of parental leave length does not alter this conclusion. In sum, the alleviating effect of family policies is only found with respect to child care support and not with respect to leave provision.^5^

In Model 2, we present the cross-level interactions as formulated in hypothesis 4. The interaction effects are non-significant, hence we have to reject our hypothesis that mothers benefit more from care and leave policies than fathers. Apparently, the gender gap in work-to-family conflict remains constant, even if welfare states provide generous support for caring families. Given that dual-earner/dual-carer related policies are often referred to as women friendly, this result is remarkable.

**Fig. 3:**
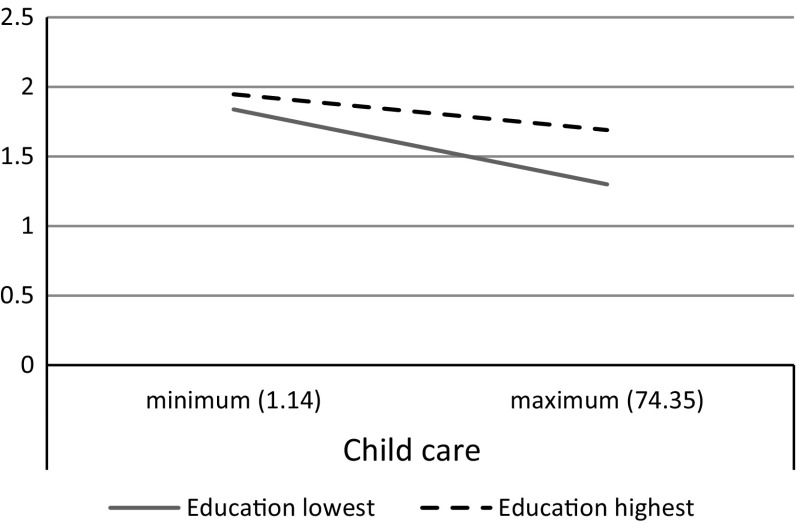
The relation between child care policy and work-to-family conflict by educational level (lowest and highest level of education)

Model 3 shows the results of the cross-level interactions with education and policy. In line with hypothesis 6, the coefficients suggest that the gap between lower and higher education widens rather than shrinks when national policies provide stronger support for child care (main effect *b* = 0.037; interaction effect *b* = 0.013). The alleviating effect of child care policies on work-to-family conflict is apparently stronger for lower educated, while higher educated parents benefit less from supporting policies (see Fig. 3). Contrasting our expectations (hypothesis 5), the results show no significant interaction effect between family leave and parents’ educational level, which may refer to the opposing mechanism at work.

In order to gain a better understanding of the gendered effects of family policies, we explore in Models 3a and 3b whether the cross-level interaction between education on the one hand and leave and care policy on the other hand differs between fathers and mothers. The results show that for men the educational gap in experienced work-to-family conflict widens as child care policies are more generous (main effect *b* = 0.030; interaction effect *b* = 0.018). However, for mothers the educational gap remains unchanged. Compared to higher educated fathers, lower educated fathers benefit more from care policies in terms of reducing experienced work-to-family conflict. Perhaps the lower levels of financial stress when two parents can stay active in the labor market due to generous child care facilities is especially relevant for lower educated men.

### Family-to-Work Conflict

Table 4 shows the results of our multilevel models estimating the impact of individual characteristics and family policy on experienced family-to-work conflict. The findings of Model 1 corroborate our expectation that mothers experience more family-to-work conflict compared to fathers (*b* = 0.068) (hypothesis 1). This seems to be in line with previous findings that working women more often take care of family tasks, and therefore experience a higher level of family-to-work conflict than men. Hypothesis 2 is corroborated as well: higher educated experience more family-to-work conflict compared to lower educated parents (*b* = 0.037). In addition, the findings in Model 1 show that people with more working hours experience higher levels of family-to-work conflict. Compared with having a non-employed partner, living with a partner who works 32–40 h a week reduces family-to-work stress, predominantly for mothers (see Model 3a and 3b). Also, especially parents with more than one child at home and living with young children (up to 3 years old) experience higher levels of family-to-work conflict (the latter primarily for women). Model 1 also comprises the country-level indicators and shows that state support for child care significantly reduces the level of family-to-work conflict (*b* = −0.102). In contrast, policy supporting maternity and parental leave has no significant effect on the level of family-to-work conflict. These results are similar to those with respect to work-to-family conflict presented in Table 3.

Model 2 estimates the cross-level interactions of family policy and gender. The findings show that the impact of policy supporting leave on family-to-work conflict does not significantly differ between fathers and mothers. This implies that support for family leave does not reduce the stress experienced due to family responsibilities interfering with work for men and women differently, which is in contrast with hypothesis 4. The results also show that the gender gap in family-to-work conflict is insensitive to policy supporting child care as well.

**Fig. 4:**
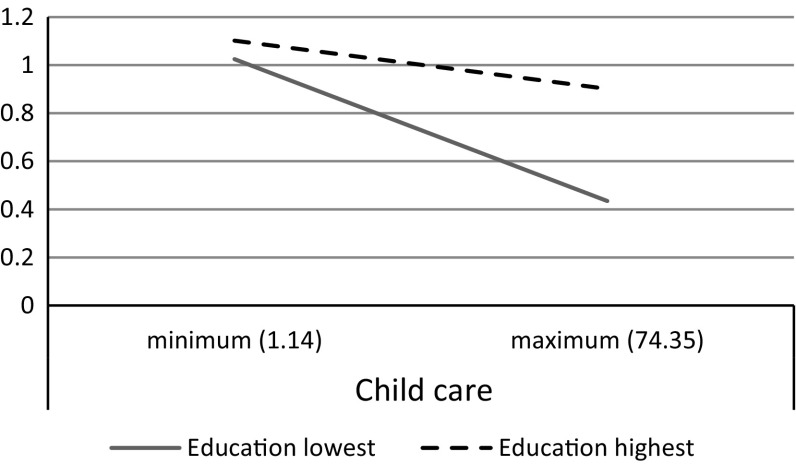
The relation between child care policy and family-to-work conflict by educational level (lowest and highest level of education)

**Fig. 5:**
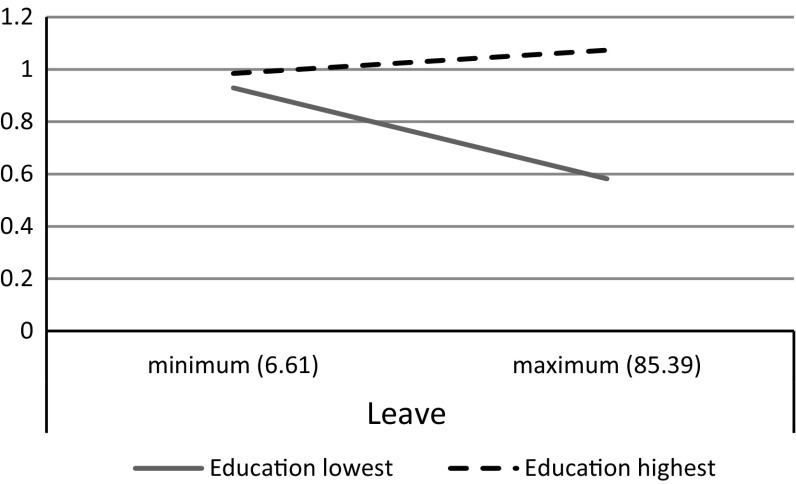
The relation between parental leave policy and family-to-work conflict by educational level (lowest and highest level of education)

In Model 3 we test whether the impact of family policy differs along educational levels. Similar to our results in Table 3, we find that the higher educated experience more conflict relative to the lower educated when their governments support child care (see Fig. 4) but also when maternity and parental leaves are supported (in line with hypothesis 6, refuting hypothesis 5) (see Fig. 5). Family policies appear to be more helpful for the lower educated as compared to the higher educated in reducing family-to-work conflict, thereby widening the educational gap in family-to-work conflict.

Modeling this association separately for men and women leads to similar findings (Models 3a and 3b), yet, especially for fathers we find that leave provision and child care are less beneficial for higher educated compared to the lower educated. In sum, the educational gaps in family-to-work conflict are larger in countries with generous family policies, especially for fathers. Future research might focus more on this relation, especially by investigating selected countries for which the distinct processes influencing the gender composition in higher and lower education, employment participation of parents and the association with family policies can be decomposed more clearly than in a multi-country study.

## Conclusion and Discussion

This study examined gender and educational differences in work–family conflict and used a comparative approach to test whether family policies, in particular support for child care and leave from paid work, are capable of reducing work–family conflict as well as the gender and educational gaps in work–family conflict. We analyzed 20 European countries with multilevel techniques using the European Social Survey 2010. We present three main conclusions and discuss their implications.

The first conclusion concerns group differences in work–family conflict. We found that mothers and higher educated respondents experience more work–family conflict compared to fathers and lower educated respectively. These findings are quite similar for work-to-family and family-to-work conflict levels. Moreover these findings are in line with the role stress theory from which we derived that women and higher educated would suffer most both in terms of time-based and strain-based conflict. Comparative time use research suggests that expectations of involved parenting as well as work demands both increased for highly educated workers (Sayer et al. 2004). Our findings inform this literature by showing that objective time demands translate into subjectively perceived stress.

The second conclusion refers to the extent to which support for child care and family leave are effective in reducing work–family conflict. Investigating the impact of specific types of family supportive policies provided important and novel insights. Most notably, not all types of policies that are usually regarded as family supportive indeed help families cope with work family stress. We found that child care support alleviates both work-to-family and family-to-work conflict. In contrast, leave policies did not affect the extent to which parents in Europe experience stress from combining work and family roles.^6^

When family policies were introduced in the European countries, the main aim generally was to increase female labour market participation while simultaneously stimulating fertility (Grönlund and Öun 2010). Others have argued that the European trend to expand leave policies served policy makers as an instrument to flexibilise the labour force (Morgan 2009; Ellingsæter 2000). Admittedly, our research did not focus on working hours or fertility behaviour, but on subjectively experienced work–family conflict. However, it is noteworthy that policies supporting temporary absence from paid work do not reduce stress-levels resulting from combining work and family roles. It has been argued that family supportive policies lead to an increasingly diverse workforce, by means of attracting more women to the labour market, implying that more couples have to combine work and family tasks, which increases the average stress levels in society (Grönlund and Öun 2010). However, this argument does not hold in the present study as it includes the group which combines work and family only. Within countries, extended leave policies are often a substitute for extensive day care provision and vice versa (compare Fig. 2). Our analyses suggest that child care support is the more effective policy when the desired policy outcome is helping families balance work and care.

The third conclusion refers to the moderating impact of family policies on the gender and educational gaps in work–family conflict. Cross-level interaction estimates showed that neither child care support nor leave policies could reduce the gender gap. These policies, often referred to as female friendly (Stier and Lewin-Epstein 2007), do not succeed in improving the position of women relative to men, leaving women in underprivileged positions. The gender gaps in work–family conflict that emerge from our models are independent of gender differences in working hours. A plausible explanation for the gender gap may be gender differences in caring hours with women spending more time in unpaid care work than men do. Moreover, higher care demands, for instance due to child birth, lead to a traditionalisation of both gender roles and ideologies (Bühlmann et al. 2010). As a consequence, women assume more responsibility and tend to be more absorbed mentally in their caring role, as compared to men (Bielby and Bielby 1989). In stimulating female labour force participation, the family policies we studied generally aimed at gender equality in participation in the labour market. In order to come closer to the ‘ideal’ of a dual-earner/dual-carer society (Gornick and Meyers 2003), our results suggest that future policies may need to be directed at increasing gender equality in both the work and the private sphere. For instance, supporting women to pursue higher occupational positions, and stimulating men to share caring tasks more equally with their wives. Examples of such policies are leave quotas in the Scandinavian countries (Haas and Rostgaard 2011).

Cross-level interactions also revealed that generous support for child care or parental leave did not lead to smaller educational differences in work–family conflict. In contrast, the low educated benefited more from extended family policies as compared to the highly educated. These findings suggest that financial incentives and job security may partially explain social differentiation in the alleviating impact of family policy on work–family conflict. We argued that leave policies may be especially beneficial for the financially better situated, as in higher educated parents. Since our findings do show that paid leave especially alleviates work–family conflict for the lower educated, this seems a fruitful starting point for future research. The findings suggest that our financial argument holds for child care but not for leave. An alternative explanation might be that higher educated parents are more sensitive to the normative message in policies that promote combining work and care as this ideal matches their ambitions and values more strongly than those of lower educated. In such countries, higher educated fathers may experience more work–family conflict because they more strongly feel pressured to be involved in both work and family roles compared to lower educated men. If this speculation is valid, it implies that it will be difficult for policy makers to reduce educational differences in subjectively perceived work–family conflict, because influencing ambitions is harder than influencing behaviour.

It is important to note that results in this study refer to working parents only and that generalisations to larger populations are not warranted. Possibly, the group we studied is selective, for instance in the sense that only people who do not suffer too much from work–family conflict remain in the labour force and hence in our sample. Some couples we observe may already have reduced conflict by postponing or limiting fertility (McGinnity and Whelan 2009). Others, most importantly mothers, may have reduced paid employment hours or taken up jobs offering lower pay in order to cope (Gash 2009). Prior research (Stier et al. 2012) indicates these issues are not likely to interfere with our results, but we cannot rule out that unmeasured traits (stress-proneness may be an important candidate) confound the relationships we find. The preferred design to deal with this problem would require longitudinal data, which are scarce in comparative research.

Another consequence of the cross-sectional design of this study is that we cannot be completely sure that policies causally affect the outcomes we are interested in. We are confident in having filtered out large parts of potential spuriousness by controlling for regime types, which can be considered proxies for countries’ overall policy context, including normative, structural, and historical features. This approach therefore produces purer effects of specific policy measures. We explicitly also acknowledge that our indicator of parental leave is ambiguous against the light of research findings that show that relatively short periods of well-paid leave encourage mothers to stay attached to the labour market, while longer periods of low-paid leave encourage mothers to stay away from the labour market for extended periods of home-care leave. In the parental leave indicator used in this paper, these two variants would obtain similar values (short period * high pay and long period * low pay). Future research may want to explicitly focus on this issue. We followed the arguments of Misra et al. (2011) and studied the role of separate policy measures instead of grouping them together in an index or categorize them in larger regimes. Our analyses show that disaggregating policies is necessary for understanding whether and how policy measures work. Most notably, our approach proved fruitful in showing distinct effects of child care support and leave policies for different social groups.

## Footnotes

1 The term family leave as we apply it in this study captures maternal, parental and paternity leaves.

2 In the ESS survey questions about work-family conflict where only asked at respondents who reported that their main activity was paid worked and who had a partner at the time of interview.

3 We chose not to include the item “How often do you find that your partner/family gets fed up with the pressure of your job?” into the work-to-family conflict scale since factor analyses showed that this item loaded on both dimensions.

4 A robustness check including job characteristics, such as EGP (class) and autonomy in pace of work and/or organization of daily work, did not interfere with our main results. As expected, it explained part of the education effect.

5 For both work-to-family and family-to-work conflict, additional analyses showed that including child care and leave policies separately reveals the same results. When leaving out regime types, the effects of the policies are in the same directions, but the effect of child-care does not reach significance, suggesting that the child care effect is suppressed by regime type.

6 Our sample includes those who are temporarily away from work. Therefore, it is unlikely that this conclusion is driven by artificially high levels of work-family conflict in the sample of countries with generous leave programmes that would result from non-observance of those individuals who supposedly experience less work-family conflict because they make use of leave programmes.
